# Supplementary material for: Usability Study of a Multicomponent Exergame Training for Older Adults with Mobility Limitations
Source: Int J Environ Res Public Health. 2021 Dec 20;18(24):13422. doi: 10.3390/ijerph182413422 (PMC8705921; doi:10.3390/ijerph182413422)
Supplement: Supplementary file 1 [file ijerph-18-13422-s001.zip › ijerph-1459331-supplementary.pdf]

International Journal of
Environmental Research and Public Health

MDPI

Supplementary Material—Usability Study of a Multicomponent Exergame Training for Older Adults with Mobility Limitations

Key Questions Interview Exergames

Overall

1. What did you remember the most? Why?
2. How did you feel while playing the exergame and why? Did you always feel the same or were there changes?

Game

1. Which game did you enjoy the most? Why?
2. Were the games structured in an understandable way? Did you have to think much about the execution of the exercises? Why/what did you have to think about?
3. Were the games easier/more difficult for you to play at some point? When (in which situation) and why?
4. How did you like the look/sound/environment/history of the games? What was good? What was bad? Why? What would you do the same or in a different way?
5. Do you have ideas/wishes for the future development of the games? What?

VITAAL Exergame/Controller

1. Did you understand the game controller quickly?
2. Were the sensors easy to use? (to put them on, left/right)
3. Could you control the games easily? What did you like the most about the game controlling? What not? Why?
4. How did you like the handling and the experience with the VITAAL exergame? Did you get along well with it? What did you like and what not? What would you do differently and why?
5. Were there any technical problems with the VITAAL exergame? If so, what were the problems and what do you think it should be like?
6. How did you get along with the game menu? What was good? What was bad? Why?

Body & Mind

1. What did you focus on in the individual games? More on the game or more on the body? Why?
2. Did the movement feel natural to you? Please describe…
3. How did you perceive the environment around you?
4. Did you feel like you were in the game? Why? All the time or with interruptions?
5. Did you always realize how good/bad you were in the game? How did you notice that?

Motivation

1. Did the game motivate you to move? How and why/why not?
2. Do you think the game is still fun after playing it a few times? Why/Why not?
3. Could you imagine using such games in addition to the traditional offer of movement therapists in the context of therapy? If so, to what extent?
4. How did you find the feedback during the game? Did you miss anything?

Training

1. What was the biggest challenge of during the training with the VITAAL exergame (balance, coordination, steps, mental activity…)?
2. What went particularly well while playing? Why?
3. Did you have to exert yourself physically? What kind of physical exertion was that? Was it appropriate?
4. Did you have to make a mental effort/think a lot? Was it mentally tiring? Why?
5. Were the desired functions (physical and mental) trained?
6. Did you feel optimally stimulated (physical and mental)? Why/why not?
7. Where did you have problems? (physical/cognitive/in general)
8. Was there something you could not do due to your (physical or mental) limitation (mobility impairment/urinary incontinence/ cognitive impairment)?
9. How did you perceive the training duration (overall)? Would you adjust the length?
10. Did you always feel safe during training? Were you afraid to fall sometimes? When exactly?

Comparison of conventional exercise therapy versus VITAAL exergame

1. How was today's training compared to your other activities and therapies in terms of effort, well-being and support? (Which option challenges you better? Which option feels better? Which option motivates you more? Why?)
2. Which catchword comes to your mind when you think of your conventional therapy?
3. And which keyword comes to your mind when you think about training with the VITAAL exergame?

Others

1. Are there any other thoughts/desires/ideas that you would like to share with us?
